# Regular Teaching Has a Significant Positive Impact on Resident Doctors’ Interest and Motivation to Pursue Orthopedics as a Career

**DOI:** 10.7759/cureus.115214

**Published:** 2026-08-26

**Authors:** Pratik Shah, Aravind Desai

**Affiliations:** 1 Trauma and Orthopedics, Diana, Princess of Wales Hospital, Grimsby, GBR

**Keywords:** career, junior resident doctors, medical education, orthopedics, teaching

## Abstract

Background

Trauma and orthopedics remains a desirable career choice for many medical students and postgraduate doctors. Multiple factors influence a resident doctor’s future career choice, including mentorship, work-life balance, culture, and clinical interest. However, regular teaching on orthopedic topics to junior resident doctors can greatly influence their desire to pursue this specialty as a career. The ability to understand orthopedic pathologies beyond those taught in medical school can significantly boost a resident doctor’s interest in the specialty.

Aims and objectives

The aim of this study was to assess whether regular teaching in orthopedics to junior resident doctors (foundation year doctors and trust grade senior house officers (SHOs)) would positively influence their desire to pursue this specialty. The objective was to monitor a change in interest in orthopedics using a 5-point Likert scale pre- and postintervention.

Methods

A survey was sent to junior resident doctors at a district general hospital, asking questions to determine factors influencing their career choice. This was followed by weekly teaching sessions on a different orthopedic topic. A subsequent survey was sent to the same junior doctors asking about the impact of the teaching on their decision to pursue orthopedics as a career. Furthermore, the resident doctors were asked whether they now considered orthopedics as a career. Results were collected using SurveyMonkey (SurveyMonkey Inc., San Mateo, CA, USA), and qualitative and quantitative analyses were performed.

Results

Six junior resident doctors completed the survey and participated in the study. There were three foundation year doctors and three SHOs. Of the participants, one wished to pursue a career in orthopedics, one in emergency medicine, one in internal medicine, and the others were undecided. Among multiple factors influencing their career choice, participants rated “Culture of the specialty,” “Role models,” and “Mentorship” as the most important. The participants also rated teaching and training as being very important in their decision to pursue a career. Teaching sessions were held on four different topics over a period of four weeks. All participants attended at least two teaching sessions. On average, three junior doctors attended each teaching session. The average interest in orthopedics prior to the teaching sessions was 2.83 (scale of 1-5; 1 = very low; 5 = very high). Following the teaching sessions, the average interest in orthopedics improved to 4.17. The effect of the teaching sessions in increasing the participants’ interest in orthopedics was rated as “extremely effective” (five of six responses) and “very effective” (one of six responses).

Conclusions

This study demonstrates the significant impact of regular teaching on the self-reported interest of junior team members in orthopedics. Orthopedic residents and consultants have the significant ability to powerfully motivate junior colleagues through enthusiastic teaching. We recommend regular teaching for junior members of an orthopedic department as an essential tool to maintain morale and bolster enthusiasm for this specialty.

## Introduction

Trauma and orthopedics (T&O) remains a highly competitive career choice for medical students and junior trainees [1]. Career selection in surgical subspecialties is driven by a complex interplay of factors, including early mentorship, academic interest, and perceived professional fulfillment [2]. While the impact of formal mentorship on specialty choice is well documented, the role of targeted, department-level educational interventions in shaping trainee career trajectories is less understood. Social Cognitive Career Theory states that clinical teaching serves not only as an instructional tool but also as a critical determinant of trainee morale and motivation [3].

Despite the importance of early specialty exposure, standard undergraduate medical curricula often lack the depth required to foster sustained interest in T&O [4]. This educational gap often leaves junior residents underprepared and unmotivated during their clinical rotations. While additional training beyond the core curriculum can stimulate interest, there is a paucity of objective evidence demonstrating how structured postgraduate teaching series impact trainee motivation. Consequently, there are often inconsistencies in teaching programs for early residency placements, which are vital for helping trainees either confirm their interest in T&O or rule it out early in their career progression.

To address this gap, this study evaluated the impact of a structured T&O departmental teaching series on junior residents' career motivation and morale. We hypothesized that a targeted educational intervention would significantly increase trainees' desire to pursue a career in orthopedics. By measuring career intent and morale before and after the teaching series, this paper provides a scalable framework for enhancing early postgraduate specialty recruitment and engagement.

## Materials and methods

Study design and setting

We conducted this prospective mixed-methods study to determine career motivations and intentions among junior residents. The study was conducted within the Department of Trauma and Orthopedics at Diana, Princess of Wales Hospital, Grimsby, UK. A total of six resident doctors participating in the rotation were eligible and invited to participate.

Ethical approval

As the study was not considered research by the NHS Health Research Authority and following consultation with the local research team, an Institutional Review Board review was not required. Administrative approval and authorization were obtained from the Clinical Governance Lead.

Participants and recruitment

The target population comprised all junior resident doctors (training and non-training grades) in the department from October 2024 to April 2025. Participation in both the instructional sessions and the associated evaluation mechanisms was entirely voluntary and anonymous.

Educational intervention

The educational intervention consisted of a four-part, curriculum-aligned seminar series covering fundamental orthopedic topics, which was delivered weekly by a senior registrar using standardized PowerPoint presentations. To maximize accessibility and accommodate clinical schedules, a hybrid delivery model was utilized, allowing learners to attend either in person or online via Microsoft Teams (Microsoft Corporation, Redmond, WA, USA). The specific curriculum spanned four core clinical domains: ankle fracture fundamentals, distal radius fracture management, shoulder instability, and slipped upper femoral epiphysis.

Data collection and instruments

**Figure 1:**
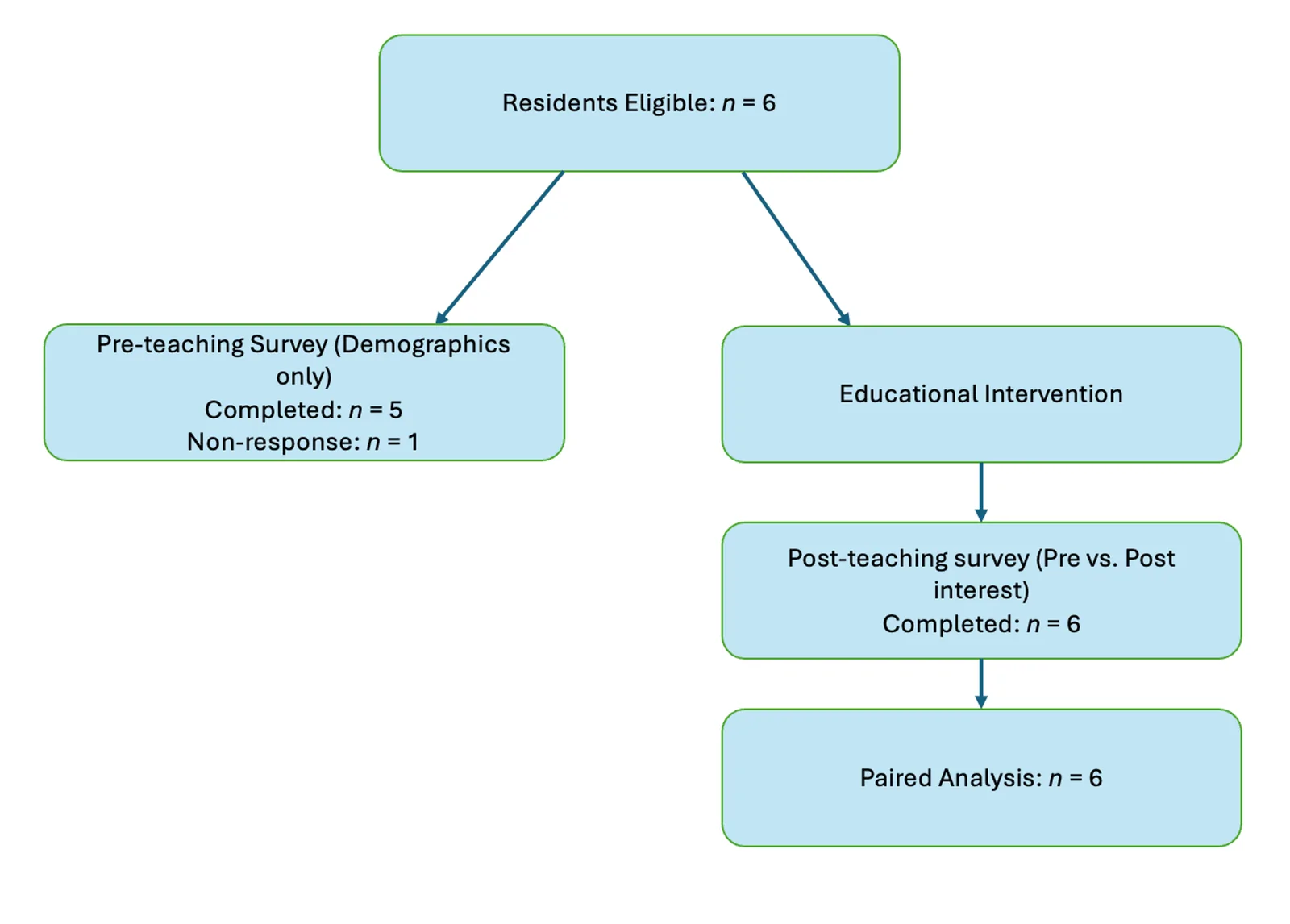
Flow diagram of resident recruitment, survey response rate, and study workflow.

Data were collected electronically using the online survey platform SurveyMonkey (SurveyMonkey Inc., San Mateo, CA, USA) across a pre- and postintervention assessment design. Data collection occurred in two phases (Figure 1).

First, prior to the teaching series, a preintervention survey was distributed to capture baseline participant demographics regarding their stage of training and identified internal and external career drivers (Appendices A-C). This initial instrument targeted specific domains, including planned specialty choices, clinical and scientific attractions, nonacademic lifestyle considerations, and the perceived value of mentorship, teaching, and departmental culture. Five of the six eligible residents responded to this baseline survey (83.3% response rate).

Second, upon completion of the entire four-part series, a final postintervention questionnaire was distributed to evaluate the direct impact of the departmental teaching on participant career interests (Appendices D, E). All six participating residents completed this survey (100% response rate). To ensure a fully matched, pairwise comparison of the primary outcome, the postteaching survey required participants to evaluate their interest in orthopedics both retrospectively (before the commencement of the teaching sessions) and immediately postintervention. Specialty interest was measured using a 5-point Likert scale (1 = “very low” interest, 5 = “very high” interest).

To monitor the delivery and immediate educational utility of the curriculum, topic-specific feedback evaluations were also collected immediately following each of the four individual seminars.

Statistical analysis

Descriptive statistics were used to summarize baseline demographic data for the subsample of responding residents in the preintervention survey. To evaluate a change in T&O interest, a paired analysis was conducted using the complete matched data from the postteaching survey (n = 6). Because of the small sample size and ordinal data, a Wilcoxon signed-rank test was utilized to compare the median retrospective preteaching interest with immediate postteaching interest. Statistical significance was defined as p < 0.05. All calculations were performed using Microsoft Excel (Microsoft Corporation).

## Results

A total of six junior resident doctors completed the educational evaluation cohort. Baseline preintervention survey responses were collected from five participants (83.3% response rate), comprising two foundation year 1 doctors (40.0%), one foundation year 2 doctor (20.0%), and three senior house officers (SHOs; 60.0%). Baseline career aspirations spanned emergency medicine (n = 1), internal medicine (n = 1), general practice (n = 1), T&O (n = 1), and undecided (n = 1). When assessing career drivers, participants identified core clinical aspects as the primary academic influence, followed by specialty role models and departmental culture. For nonacademic considerations, departmental culture was ranked as the top priority, followed by role models and work-life balance, with both formal teaching and positive specialty culture universally rated as “very important” factors influencing specialty selection.

Following the intervention, the postteaching survey achieved a 100% response rate (n = 6) from the same resident cohort. To facilitate quantitative analysis, qualitative Likert-scale fields were converted to a numeric continuum ranging from 1 (“very low”) to 5 (“very high”). A Wilcoxon signed-rank test demonstrated that the cohort’s baseline interest in T&O significantly increased following completion of the four-part series, with the mean self-reported score rising from 2.83 to 4.16 out of 5.00 (median, 3.00 vs. 4.50; W = 0, P = 0.031). This represented a large effect size (matched-pairs rank-biserial correlation, r_c_ = 1.00), with a mean improvement of 1.33 points (95% CI, 0.79-1.88) (Table 1).

**Table 1:** Participant-matched Likert-scale scores tracking interest in T&O. T&O, trauma and orthopedics

Participant	Interest in T&O preteaching sessions (1 = very low; 5 = very high)	Interest in T&O postteaching sessions (1 = very low; 5 = very high)	Individual score change
1	1	3	+2
2	2	3	+1
3	3	4	+1
4	3	5	+2
5	4	5	+1
6	4	5	+1
Mean (SD)	2.83 (± 1.17)	4.16 (± 0.98)	+1.33 (± 0.52)
95% CI	1.61, 4.06	3.13, 5.20	0.79, 1.8

Utilizing the same 5-point scale, the perceived effectiveness of the teaching sessions in enhancing the learners’ objective understanding of orthopedics was rated at a mean of 4.83, while the capacity of these sessions to actively increase their overall career interest in T&O was rated at a mean of 4.70.

## Discussion

This study demonstrates that a structured, hybrid departmental seminar series can significantly increase junior resident doctors’ interest in and objective understanding of T&O. The observed shift in mean career interest from below neutral (2.83/5.00) to highly positive (4.16/5.00) indicates that targeted local educational initiatives can actively capture the attention of undecided or flexible junior trainees during brief clinical rotations. Within modern postgraduate training, the traditional, immersive “firm-based” apprenticeship model has largely been replaced by shift-based rota systems. While these contemporary rotas have successfully optimized working hours, literature suggests that they have fragmented the continuity of surgical education, ultimately impacting junior trainee morale and intrinsic motivation [5]. Consequently, it is increasingly vital for individual surgical departments to design and evaluate novel, low-barrier educational interventions that preserve specialty exposure and restore engagement.

Our findings directly mirror contemporary medical education literature regarding specialty selection drivers. While certain international cohorts place high priority on static factors such as geographical location [6], recent data by Angeli et al. emphasize that high-quality clinical exposure and targeted education serve as the primary drivers for medical students and junior trainees when deciding their future career paths [7]. This educational influence is intrinsically linked to early subspecialty mentorship and departmental culture. In their comprehensive systematic review of mentoring within T&O, Enson et al. established that structured mentorship profoundly shapes career selection for junior residents and continues to guide subspecialty choices as they advance into senior training tiers [8]. By utilizing a senior registrar to lead these interactive sessions, our study effectively delivered micro-mentorship and created a positive departmental culture. Furthermore, this study advances the current broad-scale literature by demonstrating that large, resource-intensive infrastructure is not always necessary to garner improvement in trainee interest. We show that a smaller, targeted intervention can be equally impactful. By addressing the top nonacademic priorities identified by our cohort in the baseline survey, we achieved a unanimous increase in specialty interest (r_c_ = 1.00). This can provide a potential blueprint for low-resource departments to garner interest and motivation.

This study demonstrates how a localized, internal departmental teaching curriculum can combat modern structural training barriers to influence junior resident career trajectories. By offering a hybrid delivery model via an online platform, the curriculum successfully mitigated traditional scheduling conflicts and work-life balance concerns that trainees frequently flag as barriers to surgical careers.

Several limitations must be acknowledged when interpreting these outcomes. First, the study is constrained by a small sample size (n = 6), which reflects a single clinical department during a standard rotation block. However, despite this constraint, the cohort demonstrated a statistically significant increase in interest, with the Wilcoxon signed-rank test confirming score improvement across all participants. Because the intervention was conducted within a highly personalized, small-group setting, the scalability of these findings to larger tertiary centers, where teaching groups are naturally larger and less personal, remains unverified. Additionally, our cohort strictly comprised prespecialty trainees, including foundation year and SHOs. As a result, these findings cannot be generalized to core surgical trainees who have already declared a formal commitment to a surgical career path. Furthermore, this study evaluates a short-term self-reported increase in interest in T&O rather than long-term career aspirations; future longitudinal data are required to determine this. A further limitation is the potential for recall bias due to the retrospective nature of the survey for preintervention interest. Finally, it must also be acknowledged that there was potential for selection bias in the responses, since all participants were already undertaking a T&O placement and therefore would have had a baseline predisposition toward T&O and would not be representative of doctors not exposed to T&O. Future multicenter studies are required to evaluate whether scaling this hybrid, registrar-led departmental teaching maintains its positive influence on recruitment across larger, more heterogeneous training cohorts. We believe this methodology provided the most consistency and a direct comparison, allowing a more precise analysis of each trainee.

## Conclusions

This study demonstrates the significant positive and powerful impact of local departmental teaching on the self-reported interest of junior resident doctors in T&O. In a shift-based rota, where continuity of care and contact with mentors are often hampered, we demonstrate that a simple intervention using a hybrid platform can help break down barriers to accessing this mentorship. Ultimately, this leads to increased interest and morale, which not only serves to improve the resident experience but also increases departmental efficiency, recruitment into the specialty, and overall workplace culture. Further, larger studies are needed to determine whether these findings can be reproduced in different centers and across different specialties.

## Figures and Tables

**Figure 2:**
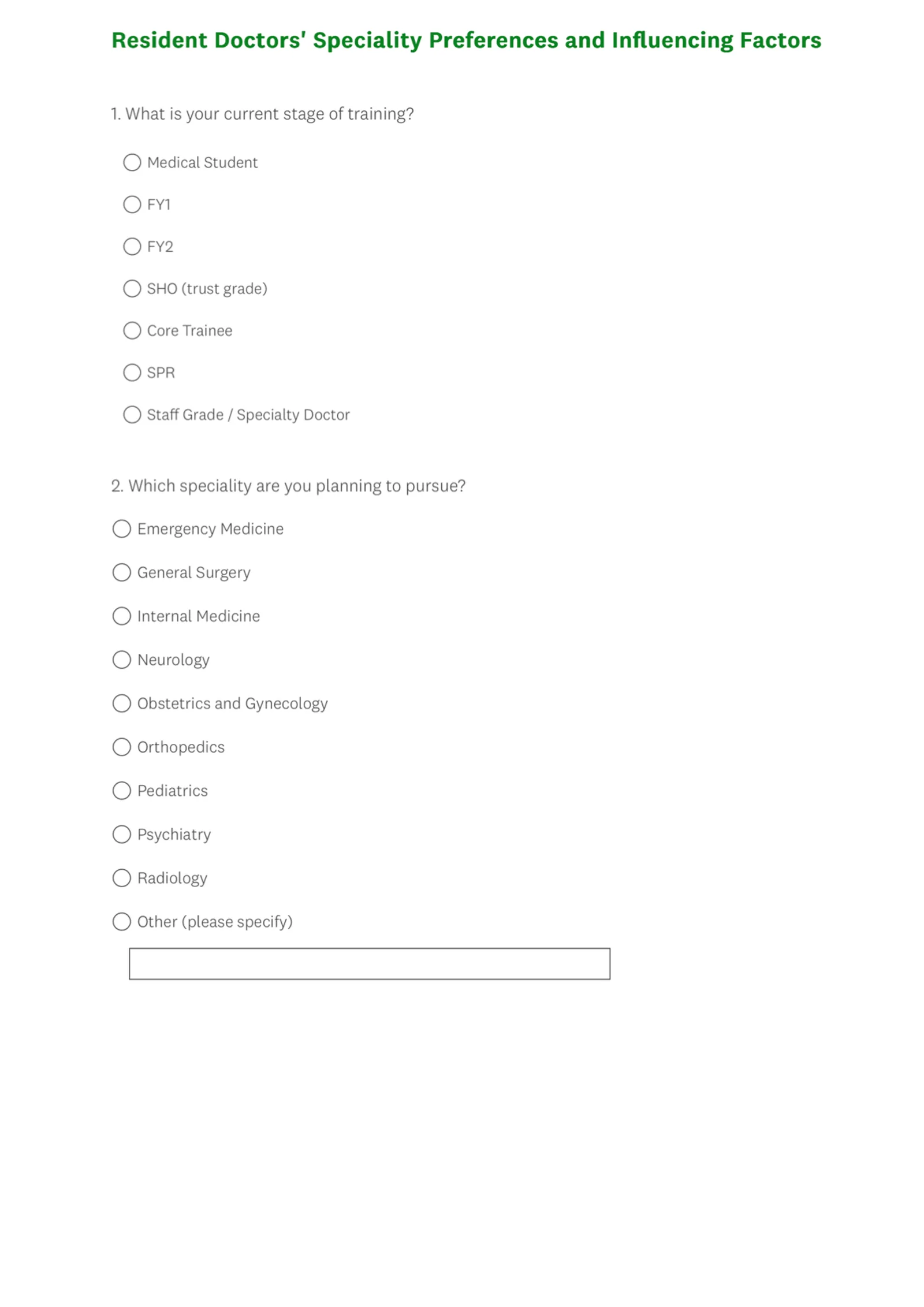
Preintervention questionnaire - page 1

**Figure 3:**
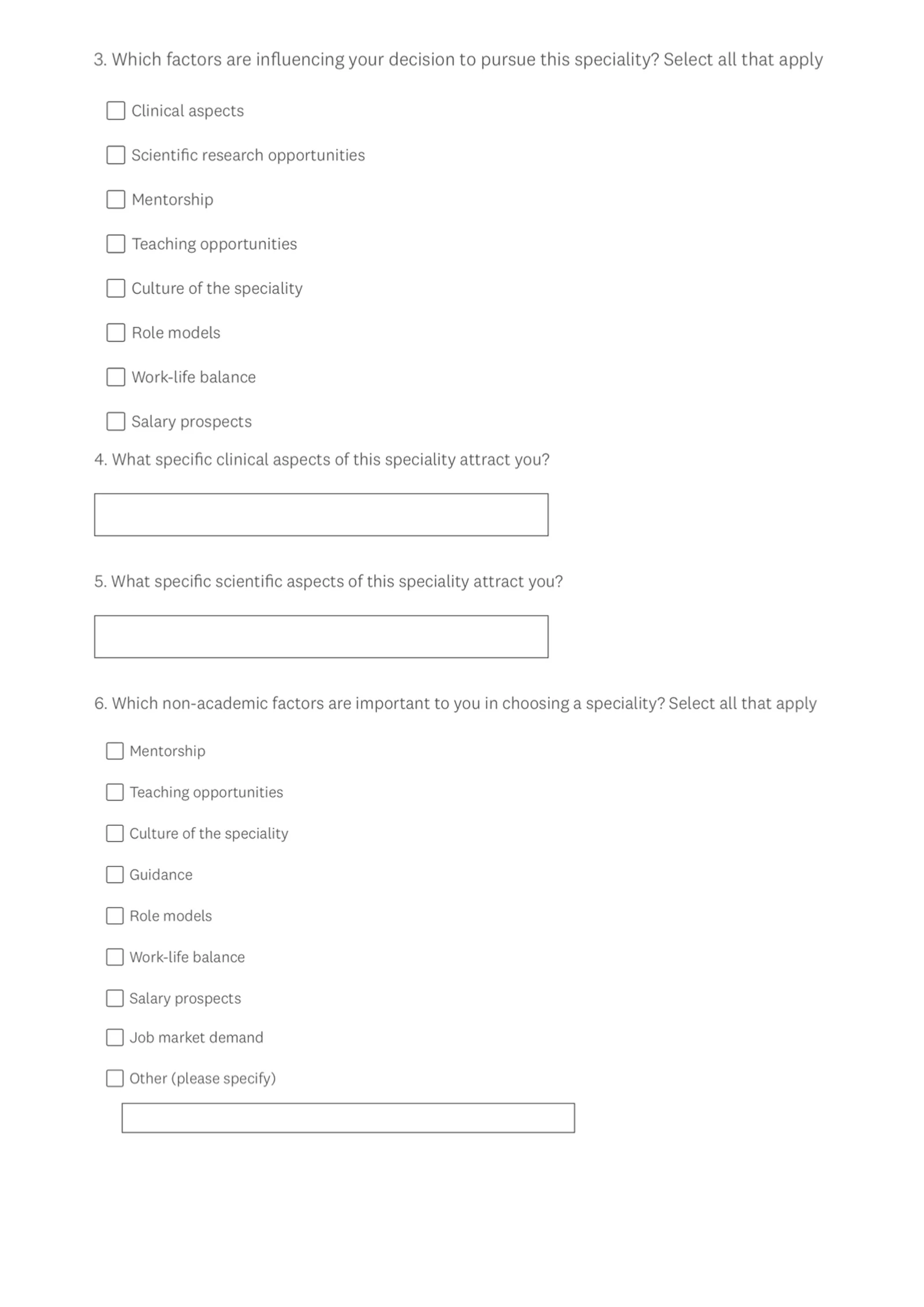
Preintervention questionnaire - page 2

**Figure 4:**
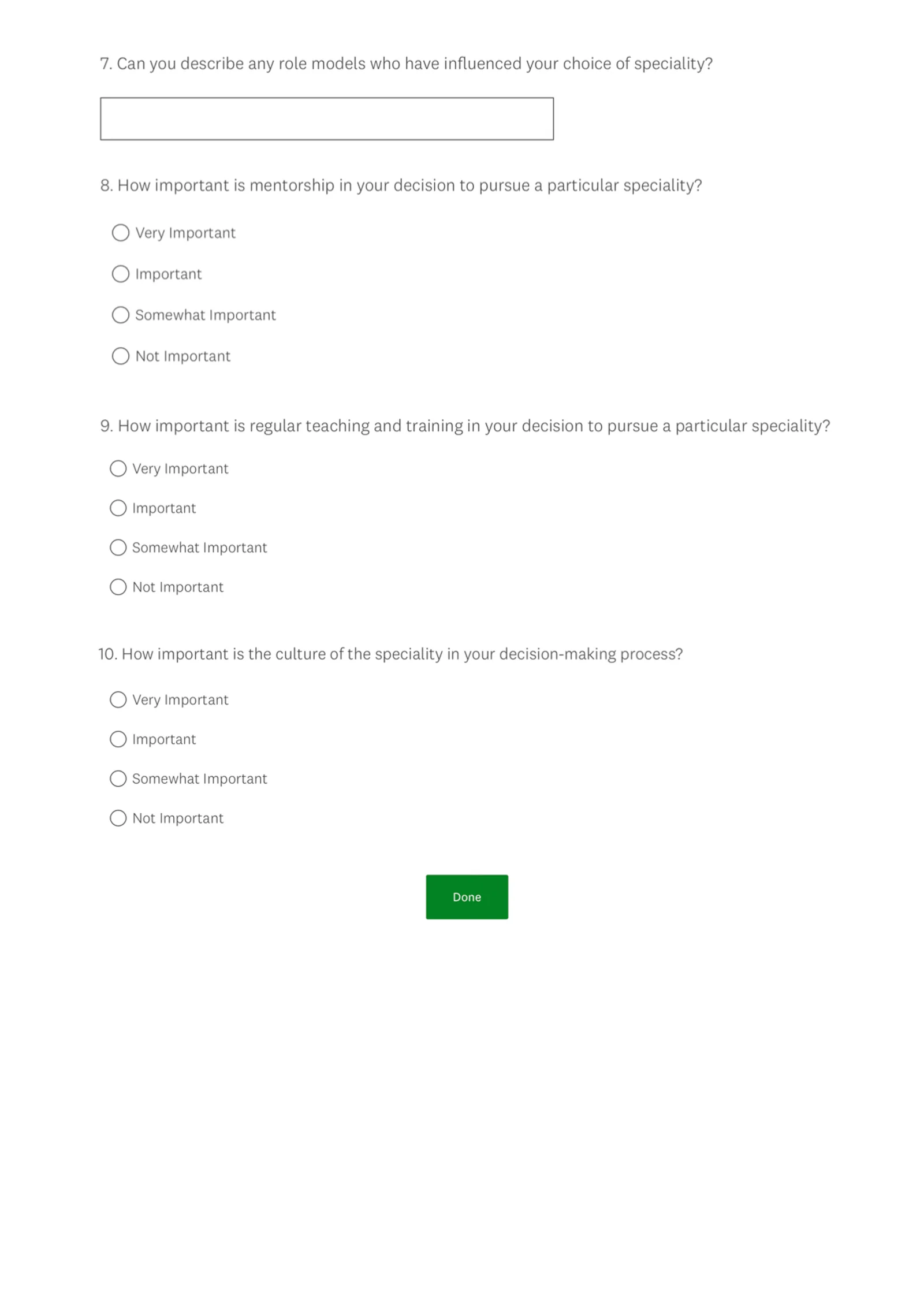
Preintervention questionnaire - page 3

**Figure 5:**
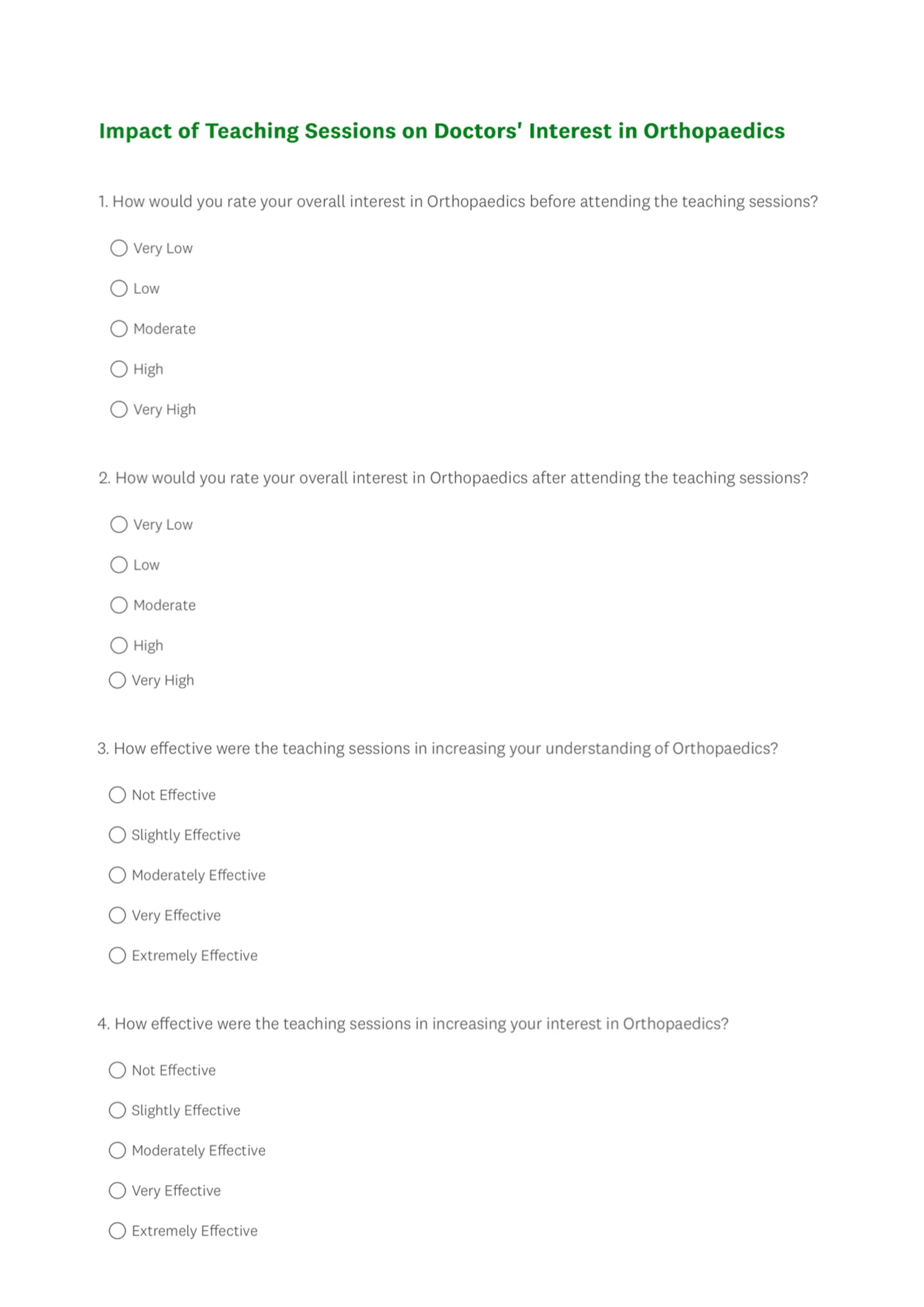
Postintervention questionnaire - page 1

**Figure 6:**
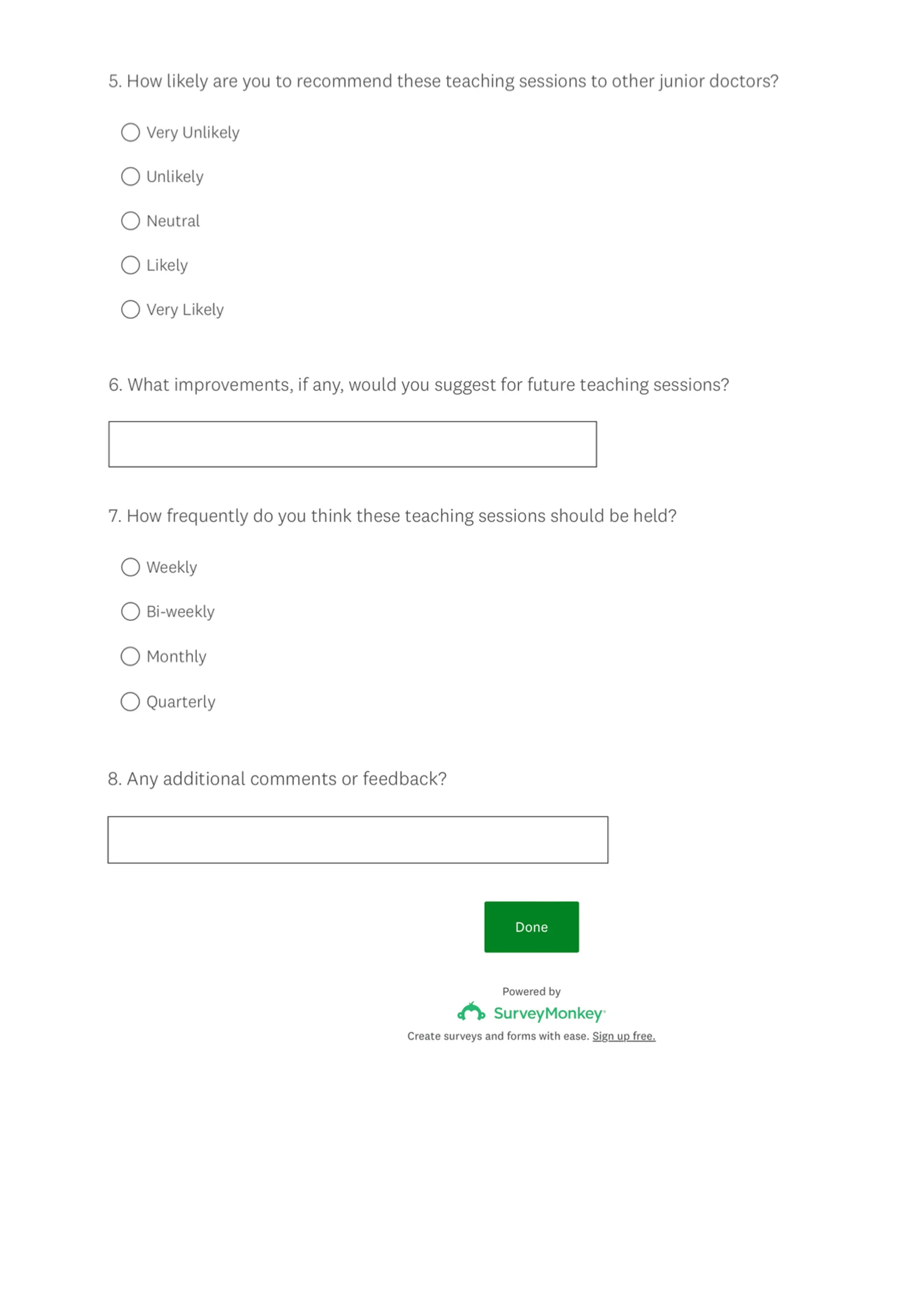
Postintervention questionnaire - page 2

## Appendices

Appendix A 

Appendix B 

Appendix C  

Appendix D  

Appendix E 
